# Psychological and Physiological Biomarkers of Neuromuscular Fatigue after Two Bouts of Sprint Interval Exercise

**DOI:** 10.3389/fpsyg.2017.02282

**Published:** 2017-12-22

**Authors:** Albertas Skurvydas, Vaidas Verbickas, Nerijus Eimantas, Neringa Baranauskiene, Margarita Cernych, Erika Skrodeniene, Laura Daniuseviciute, Marius Brazaitis

**Affiliations:** ^1^Institute of Sport Science and Innovation, Lithuanian Sports University, Kaunas, Lithuania; ^2^Department of Laboratory Medicine, Medical Academy, Lithuanian University of Health Sciences, Kaunas, Lithuania; ^3^Department of Physical Education, Kaunas University of Technology, Kaunas, Lithuania

**Keywords:** high-intensity exercise, brain derived neurotrophic factor, stress hormones, immune-metabolic response, perception

## Abstract

The main aim of our study was to determinate whether a repeated bout (RB) (vs. first bout [FB]) of sprint interval cycling exercise (SIE) is sufficient to mitigate SIE-induced psychological and physiological biomarker kinetics within 48 h after the exercise. Ten physically active men (age, 22.6 ± 5.2 years; VO_2_max, 44.3 ± 5.7 ml/kg/min) performed the FB of SIE (12 repeats of 5 s each) on one day and the RB 2 weeks later. The following parameters were measured: motor performance (voluntary, electrically induced and isokinetic skeletal muscle contraction torque, and central activation ratio [CAR]); stress markers [brain-derived neurotrophic factor (BDNF), cortisol, norepinephrine, and epinephrine]; inflammatory markers (IL-6, IL-10, and TNF-α); metabolic markers (glucose and lactate); muscle and rectal temperature; cycling power output; and psychological perceptions. The average cycling power output and neuromuscular fatigue after exercise did not differ between the FB and RB. There were significant decreases in cortisol and BDNF concentration at 12 h (*P* < 0.05) and 24 h (*P* < 0.001) after the FB, respectively. The decrease in cortisol concentration observed 12 h after exercise was significantly greater after the RB (*P* < 0.05) than after the FB. The immune-metabolic response to the RB (vs. FB) SIE was suppressed and accompanied by lower psychological exertion. Most of the changes in psychological and physiological biomarkers in the FB and RB were closely related to the response kinetics of changes in BDNF concentration.

## Introduction

Sprint interval exercise (SIE) is being used increasingly as a form of exercise training. A typical session comprises a series of brief bursts of vigorous exercise separated by periods of rest or low-intensity exercise (Buchheit and Laursen, 2013; Biddle and Batterham, 2015). Compared with traditional endurance training, the major advantage of SIE is that beneficial adaptations can be obtained with a shorter exercise duration (Bacon et al., 2013). Recent research shows that regular and optimum physical exercise plays a key role in general well-being, mental and physical health, disease prevention, and longevity in humans, and that some of these effects are mediated by the production and release of the brain-derived neurotrophic factor (BDNF) and other myokines, such as interleukin 6 (IL-6), IL-10, and the tumor necrosis factor-alpha (TNF-α) (Kramer et al., 2006; Cotman et al., 2007; Pedersen et al., 2009). High-intensity exercise improves cardiovascular health (Lowensteyn et al., 2000; Lee et al., 2011) and increases BDNF levels (Oliff et al., 1998); therefore, it may help promote synaptic plasticity and growth and the survival neurons in the brain (Neeper et al., 1995; Cotman and Berchtold, 2002), which is of great importance for overall brain heath (Marquez et al., 2015).

Sprint interval exercise is a type of exercise that should not cause skeletal muscle damage, but should cause fatigue because of metabolic stress to muscle fibers (Place et al., 2015). Regarding metabolic response, lactate has been proposed as an important glycolytically produced metabolite that is most likely released because of increased or accelerated anaerobic glycolysis and stress response. When the rate of glucose metabolism exceeds the oxidative capacity of the mitochondria (Dienel, 2013), lactate assists as a critical buffer, allowing glycolysis to produce significant amounts of energy rapidly. The acute immune-metabolic response to exercise is connected to glucose homeostasis in muscle cells (Steinacker et al., 2004; Rosa Neto et al., 2011). Acute exercise enhances IL-6 concentration by increasing its production in skeletal muscle, which acts in the regulation of the muscle energetic status (Pedersen and Febbraio, 2008). Moreover, IL-6 upregulates anti-inflammatory cytokines (such as IL-10) that prevent the exacerbation of the proinflammatory milieu, thus blocking a possible persistent inflammatory status, and downregulates TNF-α. Alterations of cortisol and IL-6 levels can regulate the availability of substrate during the exercise, and these alterations are modified by the type, intensity, and duration of the exercise (Petersen and Pedersen, 2005). In addition, possible BDNF modulation factors, such as lactate, cortisol, and intensity, have been proposed. It has been speculated that skeletal muscle cells might be involved in mediating changes in BDNF, and that skeletal muscle contractions during high-intensity exercise might be a possible trigger of a biochemical pathway linking an exercise-induced secreted factor from skeletal muscle to *BDNF* gene expression in the brain (Wrann et al., 2013). Cortisol is commonly known as a stress hormone (Sapolsky et al., 2000), and chronically elevated levels of cortisol inhibit neurogenesis and neural plasticity. More specifically, exposure to corticosterone decreases BDNF expression in the brain; this suggests a negative relationship between cortisol and BDNF (Smith et al., 1995), which alters mood and may cause depression (Brunoni et al., 2008; Stein et al., 2008; Hashimoto, 2010; Polyakova et al., 2015).

The repeated bout (RB) effect refers to the adaptation via which a single bout of eccentric exercise protects against muscle damage from subsequent eccentric bouts (Kamandulis et al., 2010; Gorianovas et al., 2013). The functional effects of applying repeated SIE on physiological and psychological biomarkers with respect to neuromuscular fatigue in healthy young adult men have not been investigated. A maximal sprint effort that lasts >30 s is associated with unpleasant outcomes, such as nausea, vomiting, and dizziness (Inbar et al., 1996). By contrast, a 5-s maximal sprint effort has been shown to be well tolerated (Verbickas et al., 2017). Considering this, in the present study, participants performed the same exercise protocol—12 repeats of 5 s—twice, once at the start of the exercise and again 2 weeks later, on the assumption that the first bout (FB) would reduce the metabolic stress on muscle fibers and the brain in the subsequent SIE bout (i.e., RB). If so, one would expect that a lower increase in lactate, glucose, cortisol, norepinephrine (NEp), and epinephrine (Ep) levels, in line with a lower increase in body temperature after RB, would promote a decreased cytokine response and a greater increase in BDNF level compared with the FB. Therefore, the participants would be more motivated to engage in exercise, would feel less exertion during exercise, and would be better oriented regarding time perception during exercise and recovery. In addition, we hypothesized that reduced metabolic stress on muscle fibers would induce a decreased release of reactive oxygen/nitrogen species-dependent ryanodine receptor 1 (RyR1) fragmentation (Place et al., 2015), thereby leading to reduced sarcoplasmic reticulum (SR) Ca^2+^ leakage and depression in muscle contractility properties and overall force production.

Therefore, the primary purpose of our study was to determinate whether RB (vs. FB) is sufficient to mitigate SIE-induced psychological and physiological biomarker kinetics within 48 h after exercise.

## Materials and Methods

### Participants

Ten physically active men (mean ± SD; age: 22.6 ± 5.2 year; height: 181.1 ± 4.4 cm; body mass: 76.5 ± 9.1 kg, and VO_2_max: 44.3 ± 5.7 ml/kg/min) participated in the current study. All volunteers were non-smokers and had no indications of cardiovascular, pulmonary, mental, or neuromuscular disease or trauma. The experimental protocol was approved by the Kaunas Regional Biomedical Research Ethics Committee and conformed to the Declaration of Helsinki.

### Experimental Design

#### Familiarization

On the familiarization day, participants completed a maximum graded exercise test to determine maximum oxygen uptake (VO_2_max) (Cernych et al., 2017) on a cycle ergometer (Ergoline, Ergoselect 100, Bitz, Germany) using a mobile spirometry system (Oxycon Mobile, Jaeger/VIASYS Healthcare, Hoechberg, Germany). Subjects with a VO_2_max < 42.2 ml/kg/min were excluded from the study based on the American College of Sports Medicine recommendation for individuals performing high-intensity exercise (Thompson et al., 2010). Each subject was familiarized with all laboratory testing procedures, equipment, psychological perception scales, and protocols to ensure that they would be able to maintain MVC of the knee extensors, to perform isokinetic knee extension, and to tolerate electrical muscle stimulation. The familiarization day was at least 7 days before the first investigation day. All participants avoided strenuous exercise for 72 h, abstained from alcohol and caffeine intake for at least for 24 h, and were well hydrated before each session.

#### Experimental Protocol

**FIGURE 1:**
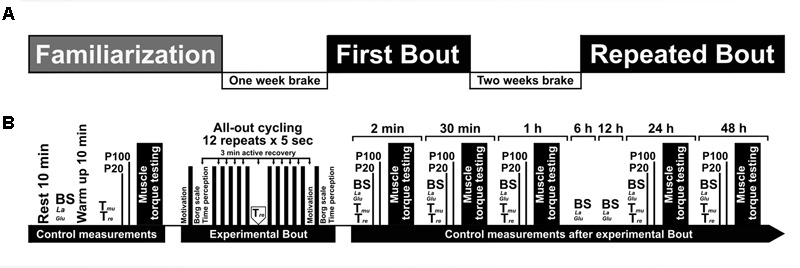
Graphical overview of the experimental protocol. **(A)** Experimental design; **(B)** protocol and control measurements. BS, blood sample; Muscle torque testing (electrical muscle stimulation; maximum voluntary contraction torque; central activation ratio (CAR); isokinetic knee extension torque at 180°/s; handgrip torque); T_mu_, muscle temperature; T_re_, rectal temperature; La, lactate; Glu, glucose.

The experimental protocol was performed at the same time of day (8:00 a.m.) for all subjects. At the beginning of the first visit, body mass was measured on a scale (Tanita, TBF-300, Arlington Heights, IL, United States), and the subject rested for 10 min, after which blood samples were taken (**Figure 1**). All blood samples were collected at the following time points: before exercise, 2 and 30 min, and 1, 6, 12, 24, and 48 h after completion of the exercise. The subject performed a warm-up on a cycle ergometer (Ergoline GmbH, Ergoselect 100, Bitz, Germany) for 10 min at a power output (W) approximately equal to the subject body’s mass (kg) and at a rate of 70 rpm. After the warm-up, body temperatures, voluntarily and electrically induced muscle torque, and central activation ratio (CAR) were measured. Muscle temperature (T_mu_) and rectal temperature (T_re_) were measured at the following time points: before exercise (exception: T_re_ was measured after the sixth series and 1 h after exercise), 2 and 30 min completion of the exercise. Knee extensor muscle torque was measured at the same times plus 24 and 48 h after exercise. Handgrip strength was measured at the same time as the measurement of knee extensor muscle torque before and 2 min after exercise; the best of three attempts was recorded.

After all control measurements were made before exercise, the subject performed a standardized warm up comprising 5 min of unloaded cycling at 60 rpm. The SIE protocol comprised 12 repeats of 5 s each interspersed with 3 min of active recovery (unloaded cycling at 60 rpm). The subject was instructed to perform a maximum effort from the beginning of the test until instructed to stop. The subject was asked to indicate his motivation level before the first and 12th interval, perceived exertion and subjective perception of work time after first and 12th interval, and subjective perception of rest time before the second and 12th interval.

Each subject performed the FB of the SIE on the first experimental day and repeated the same exercise (i.e., RB) under the same conditions 2 weeks later. This study involved several complex measurements performed at various times after SIE. After the FB and RB, the immediate priority was to obtain the blood samples and then to measure T_mu_ and T_re_, followed by voluntarily and electrically induced muscle force, and CAR testing. The time required for the blood sample collection through the muscle force testing was about 2 min.

### Experimental Measurements

#### Sprint Interval Cycling Exercise (SIE)

Cycling power output was measured during the first and the last trials of the FB and RB. Each bout of exercise comprised 12 repeats of 5 s (Verbickas et al., 2017) on a cycle ergometer (Monark, Ergomedic 894 EA, Vansbro, Sweden) interfaced with a laptop computer. Foot straps were used to secure the feet to the pedals. The pedal right arm crank starting position was 45° forward to the vertical axis. Upon the start command, the subject began pedaling and continued until the stop command. During cycling, when the subject had reached 100 rpm pedaling frequency, the computer automatically added the weight (7.5% of body mass). During the entire SIE, the subject received strong verbal encouragement.

#### Body Temperatures Measurements

T_mu_ was measured by needle thermocouple (model DM-852; Ellab, Roedovre, Denmark) inserted to a depth of 3 cm below the skin surface into the vastus lateralis muscle mid-thigh and slightly lateral to the femur. The temperature recorded at a depth of 3 cm was assumed to represent the average temperature of the active muscle mass (Brazaitis et al., 2015). T_re_ was measured using a rectal thermocouple (Rectal probe, Ellab, Hvidovre, Denmark) inserted to a depth of 12 cm past the anal sphincter. The thermocouples remained in place for 1 h after the exercise bouts. The room temperature was 21.2 ± 0.3°C.

#### Maximal Voluntary Contraction and Torque Measurements

Isokinetic dynamometer (Biodex System 3, Shirley, NY, United States) was used to measure knee extensor muscle torque. The participant sat upright in the dynamometer chair with vertical back support, and a strap secured the hips and thighs to minimize uncontrolled movement. The knee joint was positioned at a 120° angle (180° is full knee extension). MVC was reached and maintained for ∼5 s before relaxation and was measured twice, each separated by a 1 min rest, and the larger value was used in the analyses (Bernecke et al., 2017).

Isokinetic knee extension torque (IT) was measured at a speed of 180°/s (Skurvydas et al., 2010). The subject was required to extend the knee from an 80° angle. The best of three attempts was used in the calculations.

Handgrip strength was measured at the same time points as knee extensor torque using a Jamar Handgrip Dynamometer (Bolingbrook, IL, United States). The subject sat in a chair with his elbow bent at 90°. Three attempts were performed with 1 min rest between each, and the best attempt was used in the calculations.

#### Direct Electrical Stimulation and Torque Measurements

The equipment and procedure for electrical stimulation were essentially the same as previously described (Kyguoliene et al., 2017). Direct muscle stimulation was applied using two carbonized rubber electrodes covered with a thin layer of electrode gel (ECG-EEG Gel; Medigel, Modi’in, Israel). One of the electrodes (6 cm × 20 cm) was placed transversely across the width of the proximal portion of the quadriceps femoris muscle. Another electrode (6 cm × 11 cm) covered the distal portion of the muscle above the patella. A standard electrical stimulator (Digitimer DS7, Hertfordshire, England) was used. The electrical stimulation was delivered in square-wave pulses of 0.5 ms duration. Peak torques induced by a 1-s electrical stimulation at 20 Hz (P20; representing the steep section of the force–frequency relationship curve) and at 100 Hz (P100; which is close to maximum force) were measured with a ∼4-s rest interval between electrical stimulation.

#### Central Activation Ratio

The CAR was obtained during the 5 s MVC (Brazaitis et al., 2016). At ∼3 s of the MVC, a 250 ms test train of stimuli at 100 Hz (TT-100 Hz) was superimposed on the voluntary contraction. The CAR was calculated as the ratio of the maximum voluntary torque to the peak torque generated with an additional TT-100 Hz superimposed on the MVC. The 5 s MVC with the superimposed stimuli was performed twice; the larger CAR value was used in the analyses.

#### Blood Samples

Fasting blood samples were collected from median antecubital vein. Blood serum was obtained by venipuncture into vacuum tubes with a gel separator (5 ml; BD Vacutainer, Franklin Lakes, NJ, United States). The samples were allowed to clot, and the serum was separated by centrifugation (1200 × *g*, 15 min) at room temperature. Blood samples for measurement of NEp and Ep in plasma were collected into vacuum tubes with an anticoagulant (3 ml; BD Vacutainer). The serum and plasma samples were aliquoted and stored at -70°C until analysis. The concentrations of human BDNF, cortisol, IL-6, IL-10, and TNF-α were measured in serum. The concentrations of NEp, Ep, BDNF, IL-6, IL-10, and TNF-α were measured using a Gemini immunoassay ELISA analyzer (Stratec Biomedical GMBH, Birkenfeld, Germany). Cortisol concentration was measured using an AIA-2000 automated enzyme immunoassay analyzer (Tosoh Corp., Tokyo, Japan).

Glucose concentration was measured immediately in venous blood (Glucocard X-mini Plus, Japan) at the following time points: before exercise, 2 and 30 min, and 1 h after completion of the exercise. Blood lactate concentration was measured at the same times. Blood samples (0.3 μL) were taken from the fingertip and analyzed immediately for blood lactate concentration using reagent strips (Lactate Pro, Arkray, Inc., Kyoto, Japan).

#### Measurement of Perception

Motivation was assessed using a visual analog scale that ranged from 1 (not motivated at all) to 10 (extremely motivated) on a 10-cm horizontal line (Kleih and Kübler, 2013). Participants indicated their motivation with regard to the load by marking one position on the line that best represented their subjective motivation.

Participants were also asked about perceived exertion using a Borg rating scale (Borg, 1970) ranging from 6 (no exertion at all) to 20 (maximal exertion). The participants were told that they were going to exercise for 12 repeats of sprints on a cycle ergometer and that they must rate how hard and strenuous the exercise felt in the legs. They were instructed that a score of 20 on the Rating of Perceived Exertion (RPE) scale should correspond to the highest exercise load they thought they could maintain for exact sprint. RPE was recorded immediately after the first and 12th repeats.

The dimension of time is essential for everyday behavior and survival (Wittmann, 2013). The subject was not informed of the amount of work (sprint) and rest time periods during the SIE. The subject was asked to rate his subjective perceptions of work and rest time using simple visual time scales. He was asked to estimate his perception of work time from 2 to 10 s (graded in 1 s increments) immediately after each repeat. The subject’s perception of rest time was evaluated before the start of the next repeat using a scale graded in 30 s increments from 1 to 5 min.

### Statistical Analyses

The data were tested for normal distribution using the Kolmogorov–Smirnov test, and all data were found to be normally distributed. Descriptive data are presented as the mean ± standard deviation (SD). A multivariate one-way analysis of variance (MANOVA) for repeated measures was used to determine the effects of each repeated trial (FB vs. RB) on the before-exercise values of MVC, CAR, IT, P20, P100, P20/P100 ratio, body temperature, and blood markers. A two-way MANOVA for repeated measures was used to determine the effects of each repeated trial (FB vs. RB) and time on the mean cycling power output, blood markers (IL-6, IL-10, TNF-α, NEp, Ep, BDNF, cortisol, lactate, and glucose levels), body temperature (T_re_ and T_mu_), MVC, CAR, IT, handgrip strength, and electrically induced muscle properties (P20 and P100). If significant effects were found, Sidak’s *post hoc* adjustment was used for multiple comparisons across a set of conditions within each repeated-measure MANOVA. Single time-point comparisons between conditions were analyzed using independent-sample *t*-tests. In addition, an exploratory data evaluation of relationships between changes in BDNF and physiological and psychological variables was performed. Statistical significance was set at *P* < 0.05. Calculations of statistical observed power (OP, in percent) were performed, and the partial eta squared ($\eta_{p}^{2}$) was estimated as a measure of the RB task effect size. The OP for a significant effect was considered to be >80%. The non-parametric Wilcoxon signed-rank test was used to compare changes in subjective ratings of perceptions (motivation, perceived exertion, and perceptions of work time and rest time) between trials. Statistical analyses were performed using IBM SPSS Statistics software (v. 22; IBM Corp., Armonk, NY, United States).

## Results

### Effects of RB on Average Cycling Power

There were no significant changes in average cycling power between the FB and RB; the first and 12th repeats of FB were 767.7 ± 116.5 W and 782.8 ± 120.1 W (102.2 ± 9.1% of the first repeat); and RB were 786.8 ± 116.1 W and 796.9 ± 123.7 W (101.3 ± 6.1% of the first repeat), respectively.

### Effects of RB on Body Temperature

**FIGURE 2:**
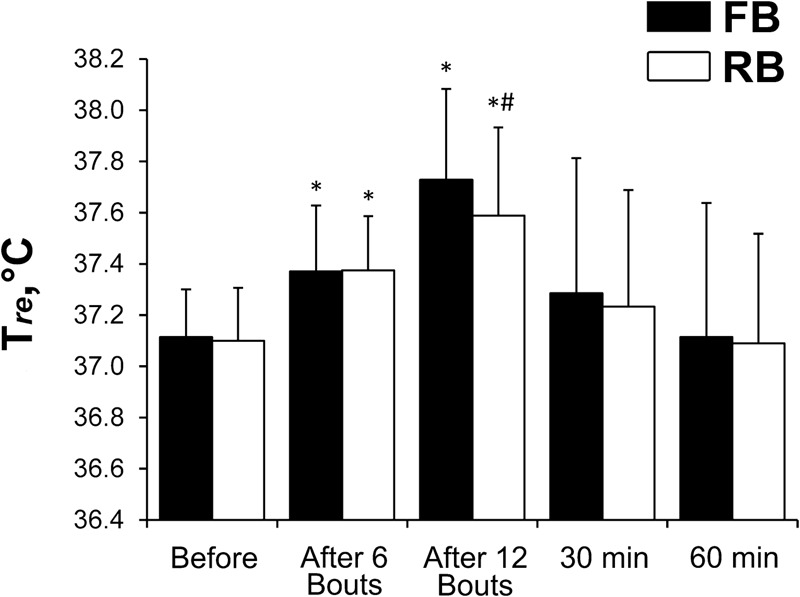
Rectal temperature changes. FB, first bout; RB, repeated bout; ^∗^*P* < 0.05 compared with preexercise values; ^#^*P* < 0.05 between FB and RB.

The T_mu_ before and 2 and 30 min after exercise was 37.0 ± 0.45°C, 38.0 ± 0.5°C (*P* < 0.001, $\eta_{p}^{2}$ > 0.9, OP > 99%), and 36.9 ± 0.4°C, respectively, for the FB and 37.1 ± 0.52°C, 37.9 ± 0.38°C (*P* < 0.001, $\eta_{p}^{2}$ > 0.9, OP > 99%), and 36.9 ± 0.47°C, respectively, for the RB. There were no significant differences in the changes in T_mu_ between the FB and RB. By contrast, the increase in T_re_ was significantly greater (*P* < 0.05, $\eta_{p}^{2}$ > 0.7, OP > 80%) after the FB compared with after the RB (**Figure 2**).

There was a significant inverse relationship between the Δ% from before to after SIE in T_re_ and BDNF (*r* = -0.74 and -0.89, respectively, in the FB and RB sessions; *P* < 0.05). The Δ% in T_re_ from before to after SIE was correlated positively with basal BDNF levels (*r* = 0.83 and 0.86, respectively, in the FB and RB sessions; *P* < 0.05).

### Effects of RB on Blood Markers

**FIGURE 3:**
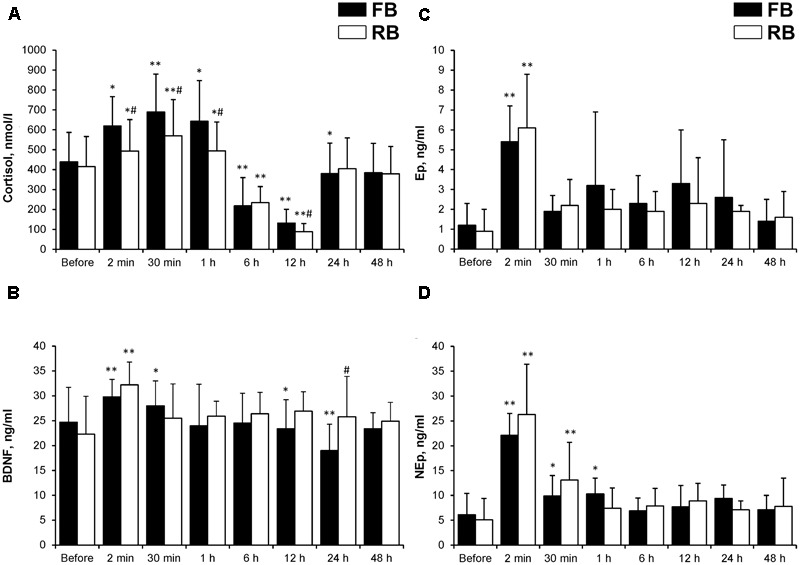
Blood indicators. **(A)** Cortisol; **(B)** BDNF; **(C)** Ep; **(D)** Nep. FB, first bout; RB, repeated bout; BDNF, brain-derived neurotrophic factor; Ep, epinephrine; NEp, norepinephrine. ^∗^*P* < 0.05, ^∗∗^*P* < 0.001 compared with preexercise values; ^#^*P* < 0.05 between FB and RB.

NEp, Ep, cortisol, and BDNF concentrations increased significantly 2 min after the FB and RB (**Figure 3**). After the FB, there were significant decreases in cortisol at 12 h (*P* < 0.05, $\eta_{p}^{2}$ > 0.65, OP > 80%) and in BDNF concentration at 12 and 24 h (*P* < 0.01, $\eta_{p}^{2}$ > 0.8, OP > 95%) (**Figures 3A,B**). The decrease in cortisol concentration observed 12 h after exercise was significantly (*P* < 0.05, $\eta_{p}^{2}$ > 0.7, OP > 85%) smaller after the RB compared with after the FB. The RB caused less stress, as shown by a smaller increase (*P* < 0.01, $\eta_{p}^{2}$ > 0.8, OP > 90%) in cortisol concentration after exercise. By contrast, BDNF concentration did not decrease 24 h after the RB.

**FIGURE 4:**
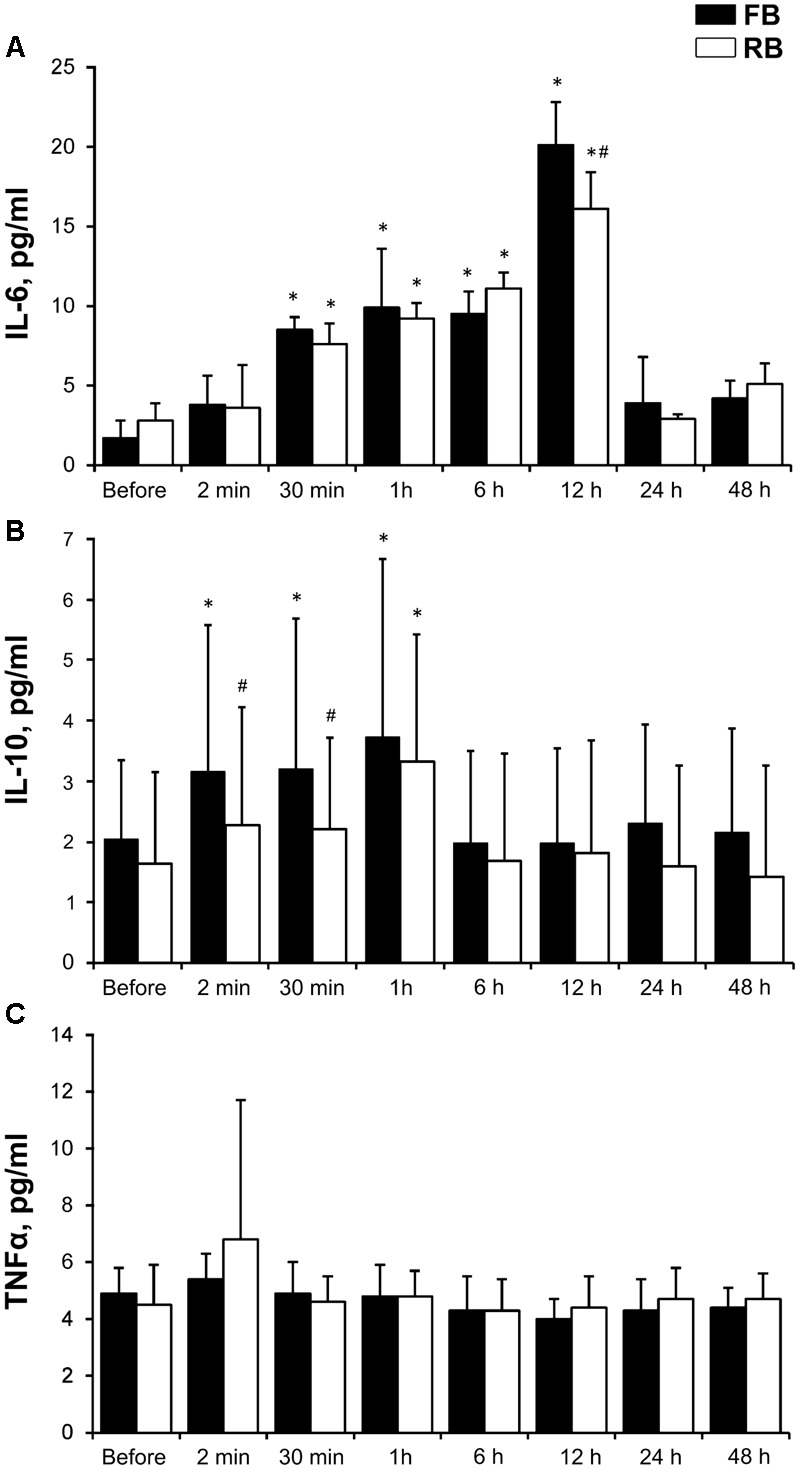
Blood indicators. **(A)** IL-6; **(B)** TNFα; **(C)** IL-10. FB, first bout; RB, repeated bout; IL-6, interleukin 6; IL-10, interleukin 10; TNFα, tumor necrosis factor-alpha. ^∗^*P* < 0.05 compared with preexercise values. ^#^*P* < 0.05 between FB and RB.

IL-6 concentration increased significantly within 30 min to 12 h after both the FB and RB (*P* < 0.001, $\eta_{p}^{2}$ > 0.9, OP > 99%). IL-10 concentration increased significantly within 2 min to 1 h after the FB and at 1 h after the RB (*P* < 0.05, $\eta_{p}^{2}$ > 0.7, OP > 80%). The concentration of TNF-α did not change after either bout (**Figure 4**). The increase in IL-6 concentration was significantly smaller (*P* < 0.05, $\eta_{p}^{2}$ > 0.75, OP > 90%) at 12 h after the RB compared with after the FB. IL-10 concentration from 2 to 30 min was significantly lower after the RB (*P* < 0.05, $\eta_{p}^{2}$ > 0.65, OP > 80%) compared with the same time after the FB.

**FIGURE 5:**
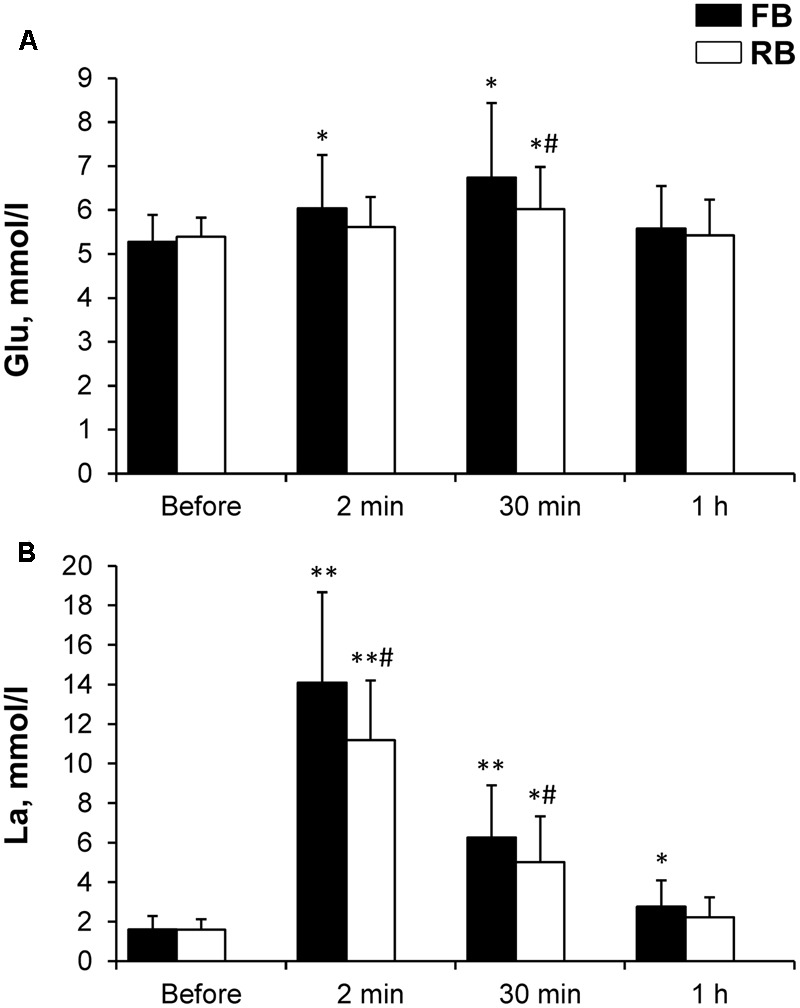
Glucose and lactate concentrations. **(A)** Glucose; **(B)** Lactate. FB, first bout; RB, repeated bout. ^∗^*P* < 0.05, ^∗∗^*P* < 0.001 compared with preexercise values; ^#^*P* < 0.05 between FB and RB.

Glucose concentration increased significantly 2 min after the FB (*P* < 0.05, $\eta_{p}^{2}$ > 0.65, OP > 80%) and 30 min after both the FB and RB (*P* < 0.001, $\eta_{p}^{2}$ > 0.9, OP > 99% and *P* < 0.05, $\eta_{p}^{2}$ > 0.75, OP > 80%, respectively) (**Figure 5A**). Glucose concentration at 30 min was significantly lower after the RB (*P* < 0.05, $\eta_{p}^{2}$ > 0.65, OP > 80%) compared with the same time after the FB.

Lactate concentration increased significantly 2 min after (*P* < 0.001, $\eta_{p}^{2}$ > 0.9, OP > 99%), and remained significantly elevated 30 min after the FB and RB (*P* < 0.001, $\eta_{p}^{2}$ > 0.9, OP > 99% and *P* < 0.05, $\eta_{p}^{2}$ > 0.8, OP > 95%, respectively) (**Figure 5B**). The increase in lactate concentration was significantly smaller (*P* < 0.01, $\eta_{p}^{2}$ > 0.75, OP > 90%) after the RB compared with after the FB.

The Δ% in BDNF concentration at 24 h was correlated significantly with the Δ% in cortisol concentration 24 h after the FB (*r* = 0.85, *P* < 0.05). We found an inverse relationship between the Δ% from before to after SIE in La and BDNF (*r* = -0.73 and -0.79, respectively, in the FB and RB sessions; *P* < 0.05).

### Effects of RB on Neuromuscular Performance

There were no significant differences in any of the muscle torque measurements at rest before the FB and RB (**Table 1**).

**Table 1:** Mean (±SD) values of voluntary and electrically induced torque before first bout (FB) and repeated bout (RB).

	FB	RB
MVC, Nm	311.8 ± 41.5	307.2 ± 44.3
CAR, %	94.7 ± 7.3	94.1 ± 7.2
IT, Nm	171.9 ± 17.7	167.4 ± 18.9
P20, Nm	181.5 ± 27.2	189.0 ± 31.9
P100, Nm	272.3 ± 30.1	270.1 ± 31.0
P20/P100	0.67 ± 0.06	0.70 ± 0.07

*MVC, maximal voluntary contraction torque; CAR, central activation ratio; IT, isokinetic knee extension torque at 180°/s; P20, torque of electrical muscle stimulation at 20 Hz; P100, torque of electrical muscle stimulation at 100 Hz.*

**FIGURE 6:**
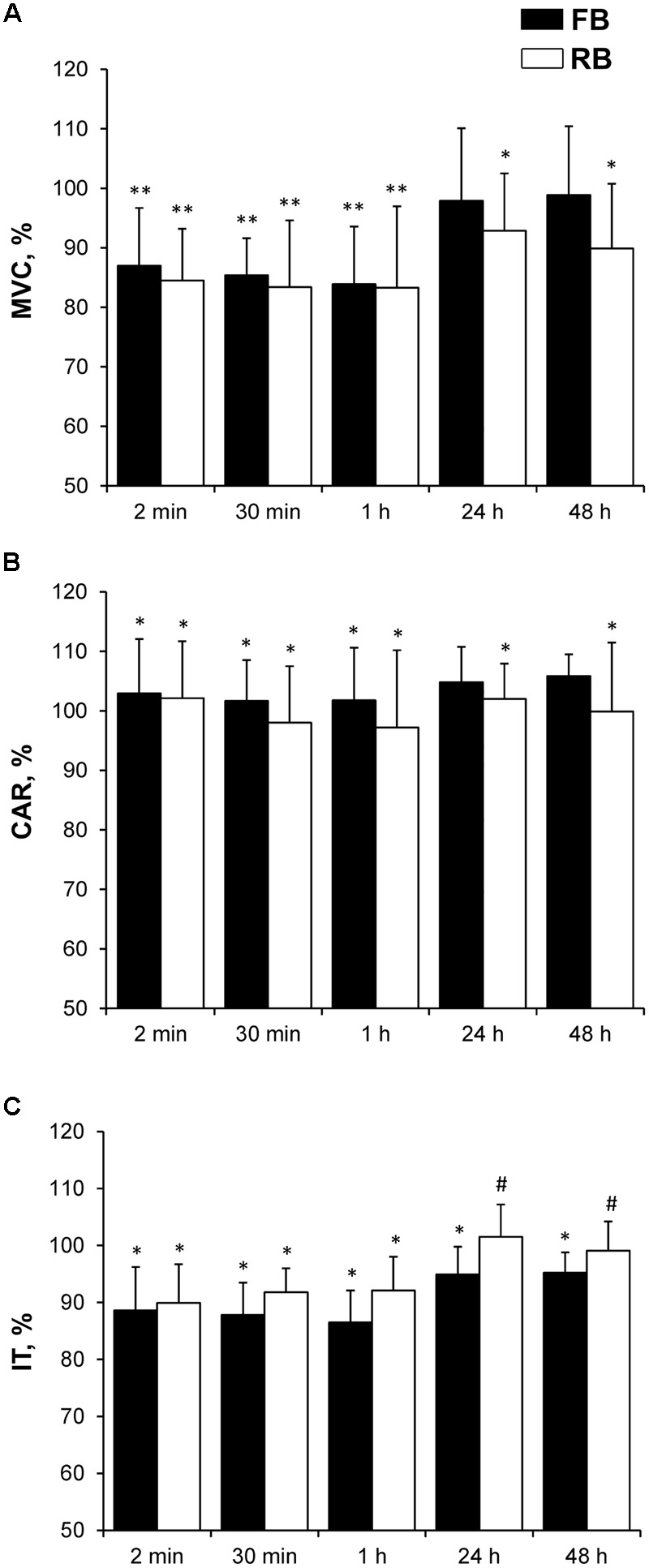
Changes in muscle force after FB and RB. **(A)** MVC; **(B)** CAR; **(C)** IT. FB, first bout; RB, repeated bout; MVC, maximum voluntary contraction; CAR, central activation ratio; IT, isokinetic knee extension torque at 180°/s; ^∗^*P* < 0.05, ^∗∗^*P* < 0.001 compared with preexercise value; ^#^*P* < 0.05 between FB and RB.

After both the FB and RB, MVC, CAR, and IT decreased significantly (*P* < 0.05, $\eta_{p}^{2}$ > 0.8, OP > 90%); however, there was no difference between the FB and RB (**Figure 6**). Recovery of IT within 24–48 h was significantly faster for the RB than it was for the FB (*P* < 0.05, $\eta_{p}^{2}$ > 0.65, OP > 80%) (**Figure 6C**). Unexpectedly, MVC and CAR recovered more slowly after the RB compared with after the FB. Twenty-four hours after the RB, the MVC and CAR values did not differ from those recorded before exercise.

Handgrip strength did not change significantly from before to after exercise: in the FB, it varied from 56.7 ± 6.1 to 57.5 ± 8.8 kg, and in the RB, from 56.0 ± 7.1 to 58.5 ± 7.5 kg. There were no significant differences between the FB and RB.

**FIGURE 7:**
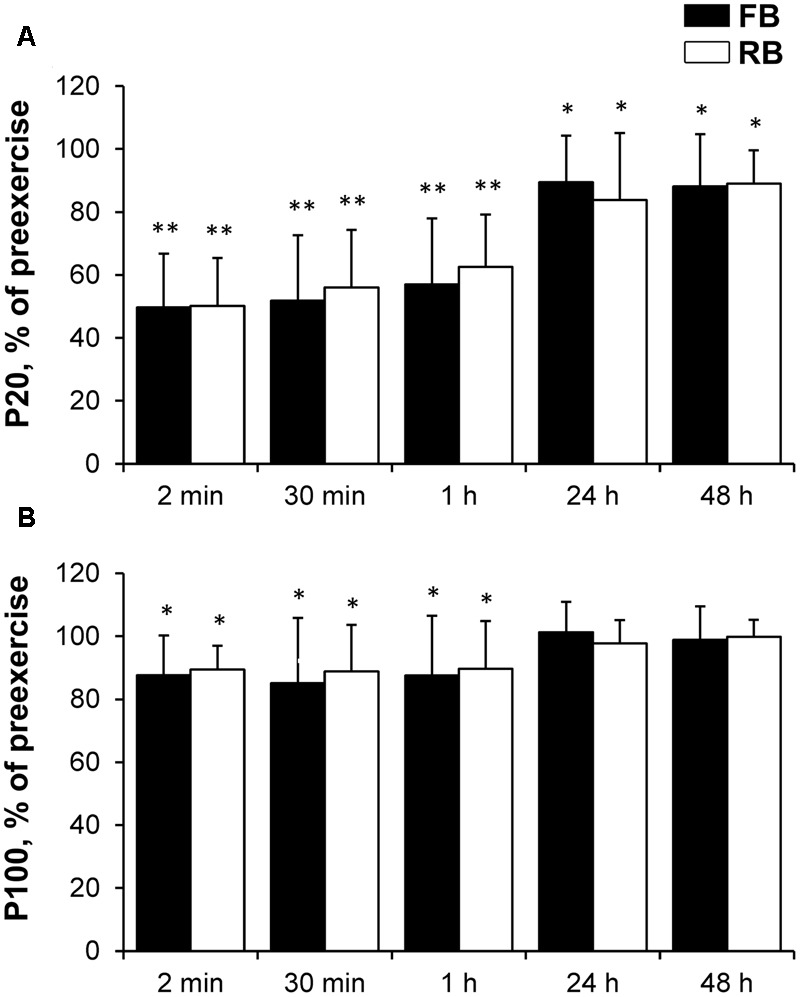
Percentage changes in electrically induced muscle torque compared with preexercise values. **(A)** P20; **(B)** P100. P20, electrical muscle stimulation at 20 Hz; P100, electrical muscle stimulation at 100 Hz; FB, first bout; RB, repeated bout; ^∗^*P* < 0.05, ^∗∗^*P* < 0.001 compared with preexercise values.

There was no significant difference in any of the electrical muscle stimulation variables between the FB and RB (**Figure 7**). The low-frequency stimulation-induced torque decreased significantly more than did the high-frequency stimulation-induced torque in both the FB and RB (*P* < 0.001, $\eta_{p}^{2}$ > 0.9, OP > 99%). The high-frequency stimulation-induced torque recovered within 24 h, whereas the low-frequency stimulation-induced torque did not recover fully within 48 h after either the FB or RB.

The percentage change (Δ%) in BDNF concentration at 24 h was correlated significantly with the Δ% in MVC and CAR 24 h after the FB (*r* = 0.80 and 0.76, respectively). The Δ% in MVC and CAR from before to 24 h after SIE were correlated inversely with basal BDNF levels (*r* = -0.75 and -0.92, respectively, in the FB session; and *r* = -0.66 and -0.67, respectively, in the RB session; *P* < 0.05). The Δ% in BDNF concentration from before to after exercise correlated positively with the Δ% in MVC from before to 24 h after exercise in the RB session (*r* = 0.76; *P* < 0.05), and with the Δ% in CAR from before to 24 h after exercise in the FB and RB sessions (*r* = 0.81 and 0.68, respectively; *P* < 0.05).

### Effects of RB on Psychological Variables

The average motivation scores were 8.8 ± 1.2 and 9.0 ± 1.1 points (*P* > 0.05) during the FB and RB, respectively. Perceived exertion increased significantly during both the FB and RB (*P* < 0.05). The average scores for perceived exertion (Borg scale) were 11.9 ± 2.8 and 11.4 ± 3.1 points for the FB and RB, respectively (*P* > 0.05 between the FB and RB) after the first repeat, and 16.3 ± 2.4 and 15.3 ± 2.1 points after the 12th repeat (*P* < 0.05 between the FB and RB).

The subjective perception of work time in the first and last repeats was 5.3 ± 0.9 and 6.4 ± 1.1 s, respectively (*P* < 0.05) during the FB, and 4.9 ± 1.1 and 5.9 ± 1.1 s (*P* < 0.05) during the RB. There was no significant difference between the FB and RB.

The subjective perception of rest time after the first and the 11th repeats was 150 ± 27 and 236 ± 43 s, respectively, for the FB (*P* < 0.05), and 176 ± 25 and 210 ± 39 s, respectively, for the RB (*P* < 0.05). There were no significant differences between the FB and RB.

The perception of work time in the 12th repeat was correlated inversely with the Δ% in BDNF concentration from before to after exercise (*r* = -0.74 and -0.72, respectively, in the FB and RB sessions; *P* < 0.05). The psychological sensations of perceived exertion during the 12th repeat and the subjective perception of work time during the FB were correlated inversely with the Δ% in BDNF concentration from before to 24 h after exercise (*r* = -0.79 and -0.82, respectively; *P* < 0.05).

## Discussion

To our knowledge, this is the first study to investigate the effects of repeated SIE on body temperature, neuromuscular functions, and markers of the immune, metabolic, and stress system in healthy young men.

In the present study, CAR decreased by about 5%, MVC and P100 by about 15%, and P20 by about 50% after both the FB and RB. We believe that the main reason for fatigue is undoubtedly associated with exhaustion of energy substrates and increased metabolite concentrations (e.g., inorganic phosphate, ADP, H^+^), which can affect the attachment of myosin cross-bridges to actin filaments, attachment strength (Allen et al., 2008), and muscle fiber activation by reducing the Ca^2+^ released from the SR (Place et al., 2015). After SIE, we found a slight but significant decrease in voluntary muscle CAR of about 5%, which returned to the initial level within 24 h. The long-lasting decrease in force observed after various types of physical exercise is more marked at low than at high stimulation frequencies and is referred to as “prolonged low-frequency force depression” (PLFFD) (Allen et al., 2008). In our study, PLFFD was apparent after both the FB and RB. The decreased force production in muscle fibers can be caused, in principle, by reduced free myoplasmic concentration Ca^2+^ ([Ca^2+^]i) during contraction, decreased myofibrillar Ca^2+^ sensitivity, and reduced ability of the contractile machinery to produce force (Allen et al., 2008). On a simplified level, the first two factors should increase the magnitude of the force depression at low compared with high stimulation frequencies because of the sigmoidal shape of the force – [Ca^2+^]i relationship, whereas the third factor should cause a similar decrease in force at all stimulation frequencies (Allen et al., 2008). In a recent study (Place et al., 2015), we showed marked PLFFD and RyR1 fragmentation in muscles of recreationally active subjects after six cycling bouts of 30 s each (Wingate bouts). Intriguingly, the same exercise caused a similar PLFFD in elite endurance athletes, but the RyR1 remained intact, and the difference could be explained by a greater superoxide dismutase expression in the elite athletes (Place et al., 2015). Thus, depending on the training status, the mechanism responsible for the PLFFD induced by repeated Wingate cycling bouts may be either impaired Ca^2+^ release from SR or reduced myofibrillar Ca^2+^ sensitivity. In this study, PLFFD did not decrease after the RB.

Acute exercise bouts have been shown to promote an acute phase response, resulting in post-exercise cytokine levels that are similar to those observed during sepsis or inflammatory disease (Nunes et al., 2015). Skeletal muscle is a major source of several cytokines, and the response is dependent on the duration, intensity, and session volume of the exercise. The cytokines have several functions and play a crucial role in energy metabolism; for instance, IL-6 and TNF-α are important in the anti-inflammatory response and exert effects on glucose and lipid metabolism, by stimulating an increase in the processes of lipolysis and glycogenolysis, to provide an energy supply for skeletal muscle and other tissues after exercise (Pedersen and Febbraio, 2008). The transient increase in the circulating levels of IL-6 during exercise appears to be responsible for a further increase in the circulating levels of the anti-inflammatory cytokine IL-10 by stimulating the release of cortisol and decreasing the levels of TNF-α. Consistently, we showed here that after SIE, the IL-10 and cortisol response initially increased progressively and reached its peak at 30 min, followed by a decrease and return to the baseline values. By contrast, IL-6 concentration increased progressively and reached its peak at 12 h. The level of TNF-α was unaffected. Such an interaction in cytokine kinetics may indicate an effect of both pro- and anti-inflammatory activity (Peake et al., 2015). The increase in cortisol concentration was a clear indicator that the SIE load caused a marked response of the HPA axis (Frx et al., 2000). As expected, in this study, the RB blunted the release of these cytokines and cortisol. Moreover, glucose concentration was significantly lower after the RB compared with after the FB; therefore, we can only speculate that the decrease in glucose concentration in the blood observed in our study reflects an increase in insulin sensitivity (Cartee, 2015).

The increase in BDNF concentration after the FB and RB in our study coincides with the findings of other researchers who reported that brief intense exercise increases BDNF concentration (Marquez et al., 2015). Shorter bouts of high-intensity exercise (1 min work and 1 min rest) are slightly more effective than continuous high-intensity exercise for elevating serum BDNF concentration (Marquez et al., 2015). The authors concluded that the SIE protocol might represent an effective and preferred intervention for elevating BDNF level and promoting brain health (Marquez et al., 2015). However, in our study, the increase in BDNF concentration was much smaller than that reported by Mang et al. (2014). Those authors showed that systemic BDNF concentration increased on average by 3.4-fold following aerobic exercise, but the changes did not relate to neurophysiological or behavioral measures. By contrast, in our study, the changes in BDNF concentration correlated strongly with prolonged changes in motor performance. Our most interesting finding is that BDNF concentration decreased gradually 24 h after exercise and that this decline was significantly greater after the FB (**Figure 3B**). We speculate that the BDNF “pit” is an indicator of a slower motor system work rate; i.e., FB was psychologically and metabolically more demanding, as shown by the greater increases in T_re_ and lactate concentration. This idea is supported by the significant correlation between the psychological feeling of perceived exertion and decrease in BDNF concentration 24 h after the FB. In our study, BDNF concentration did not decrease 12–24 h after exercise. By contrast, Ieraci et al. (2015) found that BDNF level returned to the initial value within 24 h after exercise. Rojas Vega et al. (2006) and Marquez et al. (2015) showed that BDNF level is restored to the initial level within 15–20 min after exercise. Contrary to our findings, research has shown that long-term regular aerobic exercise has a positive effect on the increase in serum BDNF level (Saligan et al., 2016). BDNF signaling mediates adaptive responses of the central, autonomic, and peripheral nervous systems to exercise (Weinstein et al., 2014), and BDNF helps to regulate energy homeostasis and energy metabolism (Pedersen et al., 2009). Our data are consistent with this concept and suggest that changes in BDNF level are closely related to changes in T_re_ and lactate concentration, although the mechanisms responsible for this association are unclear. We found that the preexercise BDNF concentration and kinetics of the change in BDNF concentration after both the FB and RB correlated significantly with indicators of prolonged neuromuscular fatigue, including central motor fatigue. That is, BDNF was a good biomarker of prolonged motor fatigue and especially central motor fatigue.

Stress is hypothesized to cause a reduction in BDNF (protein) level in the brain, which alters mood and may cause depression (Stein et al., 2008). Low BDNF level leads to a greater subjective feeling of fatigue (Saligan et al., 2016). Higher serum BDNF level may protect against the future occurrence of dementia (Weinstein et al., 2014). BDNF level may be a biomarker of mood disorders (Hashimoto, 2010) and may be a central factor in the network of multimorbidity in old populations (Krabbe et al., 2009). Low BDNF levels are found in patients with neurodegenerative diseases, including Alzheimer’s disease, and major depression (Pedersen et al., 2009). The peripheral BDNF level at rest is lower in exercise-trained people than in untrained people (Nofuji et al., 2008; Huang et al., 2014) and BDNF level increases more after acute exercise in trained than in untrained people (Knaepen et al., 2010). Taken together, these studies show that there is no consensus on the effects of exercise on the basal BDNF level and the functional significance of changes in BDNF level after exercise. The relationship between the decrease in BDNF concentration and central fatigue after 24 h has also been reported by other researchers; that is, brain performance and human well-being are associated with BDNF level (Krabbe et al., 2009; Hashimoto, 2010; Weinstein et al., 2014). It is interesting that the decrease in BDNF concentration was closely associated with the decrease in cortisol concentration after both exercise bouts. However, one may expect the opposite effect; that is, the higher the cortisol concentration, the lower the BDNF concentration because most studies show an inverse relationship between cortisol and BDNF concentrations (Issa et al., 2010; Rothman and Mattson, 2013). However, Marquez et al. (2015) did not find a negative correlation between cortisol and BDNF concentrations in humans.

BDNF inhibits the proinflammatory process (Cotman et al., 2007; Tong et al., 2008; Calabrese et al., 2014; Medina et al., 2015). However, we found no significant correlation between the changes in IL-6 and BDNF concentrations within 24 h after exercise. Physical exercise and stress can differentially modulate the expression of BDNF transcripts possibly through different epigenetic mechanisms, and this may explain why BDNF level is increased by exercise but decreased by acute stress (Rojas Vega et al., 2006). It seems that the lower initial BDNF level and the greater change after SIE should have been related to better exercise performance in our study. This idea was confirmed by the correlational analysis, which showed that the greater the change in BDNF concentration, the smaller the change in lactate concentration after exercise. Smaller changes in lactate concentration after exercise indicate higher aerobic capacity. Therefore, we speculate that a larger change in BDNF concentration after SIE may be a better indicator of aerobic capacity. BDNF is an essential neurotrophin that is also intimately connected to the central and peripheral molecular processes of energy metabolism and homeostasis (Knaepen et al., 2010). Thus, it is not surprising that the BDNF kinetics after exercise was closely related to the metabolic responses to an intensive load, as shown by the change in lactate concentration and T_re_ kinetics. The decrease in BDNF level associated with various diseases and depression (Brunoni et al., 2008; Stein et al., 2008; Hashimoto, 2010; Polyakova et al., 2015) suggest then that the decrease in BDNF concentration 24 h after exercise may be associated with increased central motor fatigue.

The psychological indicators of the prediction of BDNF kinetics after bouts seem surprising remembering the conclusions of other researchers that firstly the brain experiences the size of stress (McEwen et al., 2015). The brain perceives load size and pleasure/enjoyment very quickly and uses this information to modulate motor performance (Frazão et al., 2016). The brain is a central organ for perceiving stressors via multiple interacting mediators, including the HPA axis, the autonomic nervous system, and their non-linear interactions with the metabolic system and the pro- and anti-inflammatory components of the immune defense system (McEwen et al., 2015). Although motor performance and immune responses did not differ between FB and RB, the psychological and metabolic responses, and the stress level were lower after RB. These changes were closely related to the kinetics of the changes in BDNF.

It was surprising that the initial BDNF level predicted prolonged central motor fatigue after SIE. Other researchers have reported that BDNF level is lower at rest in exercise-trained people than in untrained people (Nofuji et al., 2008; Huang et al., 2014). We speculate that the higher training level of our subjects may have dampened the change in the CAR after the exercise bouts. Considering that the change in BDNF concentration is greater in trained people than in untrained people after an exercise load (Knaepen et al., 2010), we suggest that the greater the change in BDNF concentration, the smaller the long-term decline in the CAR after exercise.

## Conclusion

Even though the FB and RB generated similar average cycling power and caused similar neuromuscular fatigue, the stress and immune-metabolic responses to repeated (vs. FB) SIE were suppressed and accompanied by a smaller increase in T_re_ and lower psychological exertion. Most of the changes in the psychological and physiological biomarkers observed in the FB and RB were closely related to the response kinetics of changes in BDNF concentration.

## Author Contributions

The authors MB and AS contributed to the design of the work. The authors MB, VV, NB, NE, MC, ES, and LD performed the experiments. The authors MB, VV, NB, NE, MC, ES, LD, and AS contributed to the analysis and interpretation of data for the work. The authors MB and AS drafted the work for important intellectual content. The authors MB, VV, NB, NE, MC, ES, LD, and AS finally approved the version to be submitted. The author MB contributed to the revision of this work. All the authors agreed to be accountable for all aspects of the work in ensuring that questions related to the accuracy or integrity of any part of the work are appropriately investigated and resolved.

## Conflict of Interest Statement

The authors declare that the research was conducted in the absence of any commercial or financial relationships that could be construed as a potential conflict of interest.
